# Well-Being, More Than a Dream: Women Constructing Metaphors of Strength

**DOI:** 10.3389/fpsyg.2018.01186

**Published:** 2018-07-10

**Authors:** Antoni Barnard

**Affiliations:** Department of Industrial and Organisational Psychology, University of South Africa, Pretoria, South Africa

**Keywords:** women, eudaimonic well-being, psychological well-being, work-life balance, women career models, socioanalytic, social dream drawing, phenomenological hermeneutic analysis

## Abstract

Research on gender inequalities in well-being, attribute lower levels of wellness in women to the burden of multiple role demands, particularly during midlife. Using mostly quantitative measures of subjective well-being (SWB), such studies tend to narrow the concept of well-being and overlook the value of in-depth, context-specific inquiry. Work-life balance is also a consistent causative narrative in studies on women's well-being. Yet, such a narrative frequently emphasizes individual agency in a seemingly unattainable quest, implying an anomaly on how women then actually manage to sustain their well-being. The present study therefore explored the work-life experiences of women in their midlife. The aim was to reach a deeper understanding of the psycho-social dynamics at play in sustaining a psychologically-well self. Meta-theoretically the study built on non-traditional and gendered career models that augment female employees' unique career needs. From a socioanalytic stance, this study investigated secondary data gathered from focus groups, which were based on the socioanalytic method of *social dream drawing*. The data originated from four sessions of social dream drawings, in which the researcher as participant-observer, investigated work-life experiences of seven women. The gathered information was managed through Atlas.ti and processed through phenomenological hermeneutic analysis. The findings contribute to the discourse on women and well-being and give insight into the application of a unique socioanalytic methodology to research in this field from a gendered perspective. Results were analyzed by developing metaphors from the data. These metaphors reflect women's well-being as they present three unique career needs, namely challenge, balance, and authenticity, during their midlife career stage. Findings show how women's projective identification with outdated gender role norms may perpetuate a gendered notion of work-life balance, which consistently challenges their well-being. Projective identifications are evident in introjected feelings of self-doubt, self-stereotyping, and in the tension between female employees' personal and social identities. The study ultimately points to well-being as a dynamic phenomenon, which women sustain by engaging both positive and negative experiences and through identity tasks such as self-awareness, self-authorisation, self-regard, and authentic self-expression. These findings highlight the importance of creating self-reflective space in organizations to facilitate women's psychological well-being.

## Introduction

Documented evidence confirms the importance of employee well-being to deliver positive work and organizational outcomes (Harter et al., 2003; Bloom et al., 2013; Colling, 2013; Cooper and Bevan, 2014). This evidence makes the study and management of employee well-being imperative (Sieberhagen et al., 2011; Bloom et al., 2016). Despite equivocal findings on gender variations in well-being (Ferguson and Gunnell, 2016), studies show concern that women seem to be displaying lower levels of well-being than men (Dreger et al., 2016). Women, particularly during midlife, seem to face challenges to their well-being, as is evident from a recent multilevel analysis of suicide trends peaking, and overall life satisfaction decreasing for female employees during their middle age (Case and Deaton, 2015).

Most studies pointing to potential gender differences rely heavily on measures of subjective well-being (SWB) and report on statistical trends rather than provide in-depth context to explain these trends. Well-being in this regard has been assessed predominantly from Diener's (1984) perspective of hedonic or SWB, indicating a self-evaluation of the degree to which an individual experiences high positive affect, low negative affect and a high degree of life satisfaction or happiness (Deci and Ryan, 2008). This perspective, namely acting to maximize pleasure or to avoid displeasure, does not involve the pursuit of meaningful purposes in life (Ferguson and Gunnell, 2016) such as those foundational to perspectives of eudaimonic or psychological well-being (PWB) (Waterman, 2007; Deci and Ryan, 2008).

According to the eudaimonic perspective, well-being should optimize individuals' true potential and purpose in life, helping them experience their existence as meaningful, worthy, and valuable (Ferguson and Gunnell, 2016). Eudaimonic well-being presupposes self-realization and authentic self-expression (Waterman, 1993). PWB is related to eudaimonic well-being and incorporates flourishing, personal growth, environmental mastery, autonomy, positive interrelationships, life purpose and meaning as well as self-acceptance (Ryff, 1989). As such, eudaimonic and PWB perspectives acknowledge people's potential for wellness by striving for meaning and purpose, despite encountering difficulties and challenges in life. A recent study shows a decline in well-being as people age, but points out that orientation toward eudaimonic well-being, in which life purpose is maintained, can hold health benefits for older adults (Ryff, 2017).

When positive psychology was established as a new field, its criticism of psychoanalytic perspectives (Seligman, 2003) led to conceptualizing dualistic categories of well-being and ill-being (Linley and Joseph, 2004). In this regard, positive psychology theoretically rejected the negative category of human experience and negated the use of depth-psychology principles as a constructive way to study human behavior (Cilliers and May, 2010). In the present study, principles of depth psychology, as expounded in the psychoanalytic^1^ tradition, are regarded as relevant and crucial for a broader understanding of well-being. Well-being from a psychoanalytic stance acknowledges the relatedness between positive and negative human experience (Cilliers and May, 2010) and understands well-being as the manifested phenomenon of behavioral dynamics at play above and below the surface of consciousness (Clarke and Hoggett, 2009). Eudaimonic and PWB conceptualisations of well-being are therefore relevant as it provides scope for looking at interrelated positive and negative experience as fundamental to understand optimal behavior such as flourishing and personal growth.

Work-life balance is associated strongly with employee well-being (Gröpel and Kuhl, 2009; Lyness and Judiesch, 2014; Nzonzo, 2017) and considered highly important for women's well-being across their career lifespan. Studies showing women reporting lower levels of well-being, attribute this to their weaker socio-economic and power position in life and at work and to the burden of multiple role demands (Dreger et al., 2016). Thus, work-life balance can be considered as a consistent dynamic in women's attempts to maintain well-being throughout their career (Whitehead and Kotze, 2003; Poms et al., 2016). Women's empowerment and hierarchical representation in the work context is a global drive, yet traditional norms for gender roles continue to regard women as primarily responsible for domestic roles. Consequently, female employees are expected to devote less time to work than their male counterparts (Wattis et al., 2013; Sullivan, 2015; Toffoletti and Starr, 2016). As a result, women face unique challenges in work-life balance as their career progresses (Potgieter and Barnard, 2010). Furthermore, their evolving work identity and building career success differ extensively from that of men (Mainiero and Sullivan, 2005; Zimmerman and Clark, 2016).

Contemporary conceptualisations of work-life balance espouse a relativist ontology, emphasizing the attainment of work-life balance as a subjective individual experience that fluctuates over time in response to changing life contexts, career needs, identities, and priorities (Kalliath and Brough, 2008; Potgieter and Barnard, 2010; Barber et al., 2016; Kristensen and Pedersen, 2017). Such a relativist ontology and subjectivist stance developed in response to criticizing conventional perspectives on work-life balance for its objectivist epistemology of work and life as separate and mutually exclusive domains, whether it be conflictual (involving constructs such as work-life conflict or negative work-life spill-over) or enriching (using constructs such as role enhancement, work-life enrichment, positive work-life spill-over, or work-life integration) (Barber et al., 2016; Toffoletti and Starr, 2016).

On the one hand, a subjectivist understanding of work-life balance recognizes the importance of individual differences and in this study is relevant to reflect women's unique experience. On the other hand, such an understanding does not necessarily recognize the dynamics of socio-cultural and organizational contexts where individuals work and live. Instead, it places the responsibility of attaining work-life balance solely on the individual. Naturally, this creates unrealistic expectations and the additional anxiety to be an ideally balanced individual (Ford and Collinson, 2011), which applies especially to female employees (Sullivan, 2015; Adame et al., 2016; Toffoletti and Starr, 2016). Taking a psychoanalytic perspective to understanding the work-life balance dynamic, Bloom (2016) recognizes above and below surface dynamics at the intersection of the individual and the system. He highlights the elusive idea or fantasy of achieving “balance” as fundamental to the work-life balance discourse, emphasizing a never-ending and continuously disappointing quest for balance. Individuals identify and introject an imbalanced work-life identity, which provides the impetus for continued yet ultimately disappointing efforts of work-life balance in the context of corporate work demands (Bloom, 2016).

Women are therefore expected to manage the “impossible task” of work-life balance (Toffoletti and Starr, 2016, p. 490) in a socio-economic context with sustained stereotypic norms for gender roles (Burnett et al., 2010; Lyness and Judiesch, 2014). Such roles permeate organizational cultures, resulting in a gendered operationalisation of seemingly gender-neutral policies on work-life balance (Burnett et al., 2010; Ford and Collinson, 2011). To this end, female employees make multiple role transitions during a day with consequent physical and psychological costs to their well-being (Amon, 2017), especially since work-life balance remains elusive. Moreover, some women often return to careers that accommodate their role as primary caregivers (Cha, 2013; Wattis et al., 2013). Thus, they frequently opt for discontinuous, interrupted, non-linear career trajectories as opposed to the traditional career-progression patterns men tend to follow (Lewis et al., 2015; Zimmerman and Clark, 2016). Herman (2015) labels such a-typical career movements as a “frayed career” or “careerscape” to express women's range of non-typical career patterns, which include multiple career identities and career change and flexibility over their life course.

In response to the work-life balance dichotomy and due to increasing demands for work efficiency, non-traditional career models such as the boundaryless and protean ones recognize the emergence of multiple careers across organizations and time, where individuals proactively manage and reshape their career roles (Cabrera, 2009). Women applying such non-linear career models to manage work-life demands is not a new phenomenon (Mainiero and Sullivan, 2005). However, for career success, female employees are still regarded as deviating from the norm, which impedes their career advancement and hampers their well-being (Cabrera, 2009). In addition to the boundaryless and protean careers, career-path models were designed unique to women's work-life demands and disparate roles across the career life span. In light of the importance of need fullfilment for well-being, these models highlight that women's predominant career needs from their early, mid to the late career life-stages oscillate between challenge, balance, and authenticity (White, 1995; Mainiero and Sullivan, 2005; O'Neil and Bilimoria, 2005). Women's needs shift from seeking challenging work in the early career stage, to an increasing concern for balance in the mid-career and during the late career striving for authenticity and meaning (Mainiero and Sullivan, 2005; O'Neil and Bilimoria, 2005). Studies show, however, that balance remains a continuous struggle for women across generations and career stages even though it is defined differently in the various career life stages (Darcy et al., 2012; Roebuck et al., 2013).

It is evident how important well-being and women's unique career needs and their experiences of work-life balance features in a gendered socio-organizational context (Amon, 2017). Therefore, the objective of the present research was to explore the work-life experiences of women in their mid-life career to gain a deeper understanding of the psycho-social dynamics at play in sustaining a psychologically-well self.

## Methods

### Methodological orientation

Two qualitative methodologies directed the design of this study. Rooted in socioanalytic methodology, the method of social dream drawing (SDD) (Mersky, 2008, 2013) was fundamental to how data were collected. Then, data were analyzed from the methodological tradition of hermeneutic phenomenology, by employing the method of phenomenological hermeneutic analysis (Lindseth and Norberg, 2004).

Socioanalytic inquiry and hermeneutic phenomenology are epistemologically congruent, because both methodologies are fundamentally based on social constructionist assumptions. Social constructionism is a theory of knowledge or epistemology (Andrews, 2012; Galbin, 2014) emphasizing interactive meaning-making in social contexts (Schwandt, 1994) and the co-construction of knowledge between the researcher and the researched (Charmaz, 2006). Such an epistemological understanding is central to socioanalytic inquiry, which believes that knowledge is collectively generated (Mersky, 2015). Socioanalytic methods of inquiry such as SDD is further rooted in systems theory and the psychoanalytic tradition (Long, 2013). It emphasizes the collective unconscious in understanding human behavior (Sievers, 2006) and focus on group rather than individual level of data collection and analysis (Mersky, 2015). As such, the objective of SDD as a socioanalytic method, is to access collective unconscious thinking about a social or organizational phenomenon and to generate data that can be developed into working hypotheses (Mersky, 2013, 2015). The specific procedure underlying SDD is described in detail in the data collection and analysis section below and it is detailed by its developer (Mersky, 2008).

Hermeneutic phenomenology similarly emphasizes collective experience (Schwandt, 1994; Kafle, 2011) and regards the researcher as “creative contributor” central to co-constructing meaning (Dahlberg et al., 2008, p. 96). The researcher's theoretical and experiential preconceptions are central in the data gathering and analysis process, and should be transparently applied through critical and reflective interpretation (Laverty, 2003; Crotty, 2005; Dahlberg et al., 2008; Norlyk and Harder, 2010). The underlying meta-theoretical conceptualisations of well-being and work-life balance was therefore clarified at the onset of the article, it being key to the focus of the present study and interpretations of the data.

### Procedure

The researcher was involved in a project aimed at introducing and developing the SDD method for research and consulting purposes in the South African context. The original project recruited women employed at a South African tertiary institution, with qualitative research expertise and interest, to train in the SDD methodology. From the original SDD workshop several SDD sessions followed, focussing on women in the work context. The data for this article were selected from four of these SDD sessions. The inclusion of SDD data was conceptually driven by this study's espoused theoretical framework and its research objective. Four sessions included women in their mid-life career stage involved in work characterized by independent and flexible work schedules and multiple career identities typical to the “frayed” career (Herman, 2015). Purposeful sampling as defined by Yin (2011) directed the selection of these four SDD sessions as “data sources” or “sampling units” (Gentles et al., 2015, p. 1781), based on their relevance and richness to the stated research objective. Ethical clearance for using the SDD data as secondary data (because this study was not part of the original project) was obtained from the relevant institutional ethics committee at the University of South Africa (Reference number: 2016_CEMS/IOP_079). Prior to engaging in the project's SDD sessions, consent was generally obtained from participants to digitally record the sessions, as well as process the generated data for research purposes. The four SDD sessions relevant here, consisted of 14 dreams used to elicit collective thinking about work-life themes such as women working in research and the work-life role of women. The SDD data were managed with Atlas.ti and processed through a phenomenological hermeneutic analysis (Lindseth and Norberg, 2004).

### Participants

Of the seven women who participated in the four sampled SDD sessions, four women work in academia, of which two simultaneously freelance as consultants. Another participant is a professional nurse working shifts at a hospital, whilst also teaching part-time at a higher education institution. One of the participants runs her own home-based business, and the last one is involved in various non-profit activities such as writing for a local newspaper and doing developmental work in an Afrikaner Youth movement. Of the seven women, six are married; one without children and five having either two or three children. The seventh participant is a single parent with one child. All seven participants fall within the age range of 40–50. Baruch and Barnett (1986) label women in mid-life as represented by the age category 35–55, which places the participants in this inquiry within the mid-life age category. In terms of racial distribution, five white and two black women participated. Four of the women took part in three SDD sessions and three women participated in only one SDD.

### Data collection and analysis

The SDD method was developed by Mersky (2008). Approximately two to three weeks prior to an SDD session, participants receive an invitation to a themed session (in this case for example Women working in research, or Women's work-life roles). The invitation include instructions to prepare a drawing of any recent (no specific time frame given or required) or well-remembered dream. During a SDD session, participants are seated in a circle and systematically work through individual dreams according to an established procedure consisting of basically three phases (Mersky, 2008, 2013). During the first phase, the “owner” of the dream places the drawing on the ground in the middle of the circle, explains the dream drawing and answers clarifying questions from the other participants. A discussion follows in the second phase during which the other participants associate freely and expand on the dream drawing. As a method to access the unconscious, free association allows for collective reflection, drawing on the memories of participants and the social system to which they belong (Long, 2013). Thereafter and prior to the final phase, participants change seats. During the final phase, participants collectively reflect on issues emerging from the previous two phases, with the established theme in mind. Each step in the SDD process is managed according to a 15-min time boundary. Rest breaks of 15–30-min were included. As a conclusion to the SDD session, the facilitator facilitates a general concluding reflection on learnings and overall thoughts on the session and its theme as a whole. Ultimately each SDD session lasted approximately 4.5 h.

The SDD sessions were recorded digitally and transcribed. Transcriptions were loaded into Atlas.ti and a phenomenological hermeneutic analysis was done according to the typical stages of naïve reading, structural analysis, and comprehensive understanding (Lindseth and Norberg, 2004). The naïve reading and structural analysis are reported on in the results section below and the discussion section details the comprehensive understanding that emerged from the analytic process.

## Results

Through an initial naïve reading of the transcriptions, the researcher became familiarized with and gained a holistic sense of the data; in the process developing an acute awareness of the communicative power of metaphor (Shinebourne and Smith, 2010) and it being a tool to help bring to the surface emotional representations of women's well-being experiences (Grisoni and Page, 2010). The free associations and collective reflection on the dream drawings offered various metaphors. The metaphors were used to explore patterns of significance in and construct an in-depth understanding of participants' work-life experiences (Morganan, 1986; Shinebourne and Smith, 2010). During the subsequent structural analysis, several sub-themes emerged, which could be clustered meaningfully into three main themes according to the three, predominant career-development needs unique to women, namely: challenge, balance, and authenticity (Mainiero and Sullivan, 2005; O'Neil and Bilimoria, 2005). Each subtheme respectively was elucidated through the metaphors: “Icarus: Soaring dangerously”; “Centaurs: being both and”; and “Comparing notes with a stranger.” These metaphors are discussed below.

### Icarus: soaring dangerously (challenge)

In free associating to a particular dream drawing that depicts a colorful plane in the air, participants brought up the metaphor of Icarus:

*I hear the fragility, but there is also something about surviving. You know really this is like Icarus. You kept on saying that you were flying near to the edge and that went near to the sun. And you know every time you survive; you didn't fall, I mean in the dream my free association is that you are not falling out of the sky. And that you really get close to danger and this is not like slightly close to danger, this is really close to danger*.

The metaphor is derived from the Greek myth of Icarus who escapes imprisonment, yet fails because in his flight he ventures too close to the sun. As a result, the wax of his wings begin to melt so that he ultimately falls and dies. The Icarus metaphor vividly illustrates women's need for challenge but also a duality of emotions they experience in their drive to achieve career success. On the one hand, there is excitement and a sense of empowerment, on the other hand, they experience anxiety and doubt. The metaphoric scenario is the context of escaping imprisonment, which potentially represents breaking free from the shackles of entrenched gendered stereotypes that inhibit women's career success. Whereas the escape provides a sense of empowerment by achieving freedom, there is also anxiety in the prevailing gendered expectation. This is namely, that women who attempt to “fly high” are a dangerous endeavor, beset with potential failure and the imminent danger of getting hurt. The anxiety that is felt seems somehow rooted in women's own sense of “buying into” such a gendered expectation.

Discussions in the SDD groups reflected how women feel trapped despite work-life opportunities to empower them. The reason is that they work and live daily in a society entrenched with success norms that are traditionally male dominant. Thus, the participants drew on the image of a woman winning a competitive race because she “runs like a man.” Another image emerged in one of the sessions of feeling trapped like a gladiator fighting in a Roman arena. In this case, entrapment results from society's expectation that female employees will fail when they express their drive for achievement, competition and need for challenge. As one participant explained: “That makes me also think you know, this Gladiator movie and the coloseum and being trapped inside. And not able to get out, because the steep stairs only lead down, but she never reaches the bottom.”

The participants also reflected on how their needs for achievement are set up for failure by their own doubts:

*Is it safer to stay with the crowd and not stand out by choosing the easy road, even if you know that might be the best thing for you, because you are tiny? And, ja, the word that comes to mind is around self-sabotage. Because…you choose to stay with the crowd, almost intuitively knowing you are never going to get anywhere*.

It seems that women's need to belong may perpetuate their projective identification with society's entrenched expectations about gender roles. In this way, “standing out” (achievement/success) is self-sabotaged in favor of metaphorically staying with the gendered norms and low achievement expectations of the crowd. Thus, ultimately in this dynamic inner struggle between wanting to achieve and wanting to belong in a gendered society, women find their well-being challenged.

The same inner dynamic also helps build resilience where women take on the challenge of working and living in a gendered society. Thus, they embrace both their “fragility” and their “surviving”, thereby reframing the outcome of the Icarus narrative into non-failure. As a participant explained: “Every time you survive; you didn't fall…you are not falling out of the sky.” Resilience evolves when women develop a self-awareness of the need to belong and be accepted, which potentially may be fundamental to self-doubt. As attested by a participant: “It relates to the fear of being acceptable to others…and my own doubt, let me be honest about that, my own doubt in what I do.” Such self-awareness seems fundamental to well-being as it creates a willingness to take responsibility for overcoming intra-personally entrenched gendered stereotypes. The self-awareness leads to self-authorisation to take on challenges and be successful:

…*myself as a woman can I dare to break away from the crowd…because the moment that you break away from the crowd, that is when you will be, I mean you can get into serious trouble. I understand that, but that is also the time that you will be celebrated beyond imagination…if you run and you succeed, do you actually, do you actually allow yourself to be successful?*

### Centaurs: “being both…and…” (balance)

The metaphor of the Centaur depicts the phenomenon of work-life balance as more than consciously managing multiple roles within a limited timeframe. The women are thus associated with Centaurs: “I thought you drew those horse people…horses with human bodies and heads like the ones in the movie Narnia.” In this regard, they embody work-life balance by containing the psycho-social consequences and gendered expectations linked to the variable roles in their lives. Stated differently, they embody the gendered rhetoric that underpins conventional conceptualisations of work-life balance. Related associations to the Centaur metaphor are women compared to being “both fish and bird” and “both an airplane and an insect.”

Centaurs, the half-human, half-horse. creatures from Greek mythology are regarded as liminal beings. Thus, they embody contrasting natures and occupy anomalous or dual positions at the same time (Clauss, 2007). Women often find themselves in a position that simultaneously presents mutually excluding as well as conflicting expectations. On the one hand, they fullfil the traditional role of mother and wife, which is presented as sub-important to the so-called full-time working role. Thus, by implication the maternal role is also sub-important to that of male employees who continue pursuing hierarchical and financial career success. On the other hand, women fullfil a work role that is loaded with expectations to be productive and to utilize the emancipatory and empowering opportunities available for women in the workplace. As one participant stated: “Linking with that again the whole thing about contradictions, being both an airplane and an insect all at once. Being both strong and vulnerable, both agile and quite sluggish.”

Fulfilling differential roles with conflicting expectations, represent above-surface behavioral dilemmas that reflect typical issues of work-life balance and managing of time and resources between different roles. However, in-depth reflection on the embodiment of their contrasting natures by using metaphors and free association, revealed below-surface dynamics. This particularly applies to women's preoccupation with maleness, as the following excerpt illustrates:

*I'm just wondering about our pre-occupation with the penis. Because it appears after and in our association with the drawings … we are pre-occupied with men. It is almost as if the men are drawing themselves into our dreams … Excuse me, we draw them into the dreams*.

The preoccupation was given meaning during the SDD sessions, where participants split male and female qualities stereotypically. The participants projected onto maleness the characteristics of strength, leadership, and control, while femaleness was depicted with comparatively smaller feats: “In the plane you are not the pilot. But with the insect you are steering, you are controlling where you are going” and “one might think of the men being the pilots.” Due to this preoccupation with maleness, it seems that women unconsciously avail themselves to embody the gendered rhetoric that underlies the discourse on work-life balance. According to this rhetoric, men are privileged by having more career opportunities and less concerns with work-life balance. A participant elaborated: “The women is in darkness and the men know everything…the only opportunity for seeing what is out there is at the tail end and only the men seem to have access to open that space.” Women's well-being is thus challenged by their limiting perspective of balance as a gendered issue.

Nevertheless, ultimately the Centaur metaphor emerged as an image of strength. Female participants also identified within themselves a reservoir of both maleness and femaleness: “I like that about her because I like to be male and female. I really like that. I don't like only the one…because what she then brings to my mind is that she holds the ambivalence inside…” In being “both … and …” women see the potential for different rules of being in the world. This insight is described by the following excerpt:

*For me it is almost like a fish crossed with a bird and I know you get these movies—“Journey to the center of the earth,” where the rule is everything is the opposite than normal. So, the elephants are small where the insects are big; and you get, let's say, ducks with a buck's head or a fish with bird's wings and the bird's beak*.

By understanding the possibility of different societal norms there is also the potential to forge new constructions and meaning. A participant remarked: “So, maybe it's about male and female, you know. Maybe it's not just about male…like I mean, for me the courtyard now is a space for something to be birthed.” Women's well-being in this case, seems to be their embodiment of difference and their capability to in fact assume a liminal position. This may be on the boundary of work and life where they present both male and female qualities.

### Comparing notes with a stranger (authenticity)

An interesting dream shared in one of the SDDs, illustrates two figures sitting back-to-back each with religious notes on their laps. The participant titled her dream drawing, “comparing notes with a stranger.” She identified herself, a Christian woman, as one of the figures sitting with her back to that of an unknown Islamic man. In the dream she experiences surprise and relief when realizing that the notes on his lap are similar to her religious notes: “I looked over and said, ‘Hey it's the same’ … and it was appropriate.”

“Comparing notes with a stranger” metaphorically reflects the tension that women experience when they attempt to retain their personal identity and their authenticity, in relation to their gendered social identity (Kreiner et al., 2006; Brown, 2015). The dream carries a strong spiritual theme. It speaks to the core of one's being and expresses what one believes, relating to the need for authenticity and a distinct personal identity. This dream metaphor expresses how women experience their gendered social identity through negative emotions: feeling judged and not being good enough. The stranger resembles the object of badness (projected onto him) and how the woman identifies (introjection) with the good and preferred characteristic of being non-judgemental. The projection of badness and participants' response to it, represents women's expressed need to be accepted and not be judged:

*Central to the dream is something I believe very strongly in. One shouldn't judge. I feel there are people in my house that are terribly judgemental. Just like that, judge, judge, judge, and it's something I've felt my whole life: I can't judge others. I just immediately felt this guy, maybe from ISIS or whatever, I can't judge him*.

Attempting to maintain a personal identity of competence, women find it challenging to reconcile a competent self with stereotypic gendered expectations of performing inferior work. Confirming this dynamic is another dream in which one female participant receives flowers from her husband's work, while she is at a workshop: “I didn't know it was for me; it was very weird. I thought the flowers came from my husband's work and it was extremely weird that they knew I was at the workshop.” Women's need for recognition as being competent seems evident in the dream, yet it is conflicted by their experience that others do not expect them to be productive: “I couldn't understand that they knew I was there and not at home.” The tension between the personal identity and the gendered social identity was first recognized where women feel judged. The tension was affirmed in this dream where the participant had a sense of not being valued.

To resolve and balance the conflicting expectations between their personal and social identities, women frequently assess their self-worth through constant interpersonal comparison. This is typical to conceptual definitions of identity work (Adams and Crafford, 2012). Participants attested that they frequently were negotiating who they are (personal identity) in comparison to others and others' expectations for them (social identity). Reflecting on her interaction with a group of young adult women at a wedding, a participant stated:

*I wish I was as sorted out as they are at that age … they know what they want and how and what; this girl is 24 and she knows exactly what she wants. I mean, when I was 24 everything was confused and then I think: ‘Wow! I am actually not even where she is now.’ She is spiritually sorted*.

Another participant affirmed: “You know our family is unstable, and then I think yours always looks so sorted.” From such a discussion comparing oneself with and idealizing others, it seems evident that women's well-being is challenged by the tension created between their sense of self and of an idealized social construct of self. The idealized self is rooted in their gendered identity and their beliefs about the gendered expectations flow from that identity.

The dream, “comparing notes with a stranger,” ultimately demonstrates how women sustain their authenticity and well-being by working across differences. This movement is reflected where women integrate the projected bad and introjected good in themselves. In the case of the dream, the woman ironically identifies strongly with the stranger: “He was totally strange to me, but I felt an immediate connection with him.” By embracing difference, women accept the good and the bad within themselves, expressing the need for an integrated, authentic self. Furthermore, women experienced relief as a strong emotion when they realize the similarity of core beliefs across above-surface differences such as gender and religion. This identification of similarities that transcend differences, reflects the identity task of integration and expresses self-regard.

## Discussion

The aim of this inquiry was to reach in-depth understanding of the fundamental psycho-social dynamics explaining how women in their mid-life career sustain well-being. This discussion details the comprehensive understanding that resulted from the analysis and includes a reflection on its implications, potential limitations and directions for future research.

### Comprehensive understanding

Career needs unique to women's careers are: challenge, balance, and authenticity (Mainiero and Sullivan, 2005). All three needs emerged in the data as persistent career issues that impact participants' well-being dynamic. In the discussion above, balance was found to be important across the career life-stages of women (Darcy et al., 2012; Roebuck et al., 2013). In this case, the data provided convincing evidence that in addition to balance, challenge remains a need women have to contend with in mid-life, while authenticity is already a relevant issue in this career stage. The results thus demonstrate that, apart from balance, challenge and authenticity needs are also prominent in the mid-life career stage and are not exclusive to women's early and late career life-stages. The present study did not aim to compare the extent to which these needs manifest in different career life stages. The focus was rather on the psycho-social dynamics at play when these women deal with their need for challenge, balance, and authenticity to sustain their well-being in work and life.

Regarding the need for *challenge*, well-being is evidenced in women's need for purpose and self-optimisation. However, at the same time, their well-being is challenged by introjections of self-doubt and anxiety, which participants described. These impediments arise from their struggle with internally entrenched gendered stereotypes and identification with masculine norms of success. Women's need for challenge is paralleled by their psycho-social need for belonging, the latter prompting projective identification with gendered expectations about career success and achievement. This detrimental identification is thus exacerbated by a conflicting dynamic that underpins the women's fundamental psycho-social needs. These needs are striving for achievement and to stand out, weighed against wanting to belong (Knez, 2016). In the same vein, Grisoni and Page (2010) report ambivalence about their competence to be rooted in their need to succeed being in competition with their need to collaborate.

Similar to challenge, the gendered notion of work-life *balance* is linked to enduring values and beliefs relevant to the socio-cultural context of work life. The findings of the present study have shown that the gendered notion of work-life balance is also perpetuated through women's projective identification with its underlying outdated norms for gender roles. Thus, women are not only sustained by society but sustain themselves by containing the gendered role expectations in work-life balance. Evidence in this regard is participants' preoccupation with male privilege and superiority as well as the stereotypic splitting of male and female qualities. This self-stereotyping prevents women from flourishing and impedes autonomy. Self-stereotyping feeds the prevailing gendered perspective of work-life balance and hampers them in sustaining their psychological well-being.

Finally, regarding the need for *authenticity*, it became evident that conflicting role expectations impact women's well-being by challenging their identity work. Identity work entails attempts to reconcile the tension between preserving the self (personal identity), and external expectations and role demands (social identity) (Kreiner et al., 2006). This reconciliation is key to well-being since it highlights the need for authenticity or the ability to express the inner self and engage in activities that reflect the true self (Vleioras, 2005; Vleioras and Bosma, 2006). The process guiding identity work strives to balance the tension that underlies the psycho-social needs of differentiation (negotiating a unique self) and integration (associating the self with others) (Adams and Crafford, 2012; Knez, 2016). Participants' identity work revealed extensive challenges to their well-being since their social identities entrenched negative connotations to traditional female roles set against their personal needs for recognition, value, and competence. In their autoethnographic research, Grisoni and Page (2010), (p. 22) similarly reflect on their having “internalized the rational male gaze from the organization, and when confronted with it we were unable to sustain a sense of the value of our own enquiry.” Such entrenched gender role stereotypes inhibit authentic self-expression or “personal expressiveness” (Ferguson and Gunnell, 2016, p. 429; Waterman, 1993, p. 678) and leaves women feeling judged and under-valued despite attempts to realize their career potential and express their personal identities.

When examining the three metaphors, it seems that women's projective identification with stereotypic gender roles may set up the primary intrapersonal barrier to their well-being. This is firstly, evidenced in self-doubt encouraged by the introjected belief that it is dangerous for women to respond to challenges due to the high potential of failure. Introjecting gendered role norms result from women's need to belong, yet conflicts with their need for challenge. Secondly, this barrier became apparent when participants split male and female qualities, where the male represents a projected superior competence in comparison to the female. Splitting of male and female qualities lead women to self-stereotype and embody the gendered rhetoric underlying work-life balance. Lastly, women's struggle to reframe their stereotypic gendered social identities, highlights the difficulty to express an authentic personal identity congruent to the need for challenge, recognition, success, belonging and acceptance. While formulating an identity, the self is continuously negotiated in relation to a socio-cultural and organizational context (Adams and Crafford, 2012). Thus, the self becomes a product of a socio-cultural past and its entrenched memories (Knez, 2016). It is therefore not surprising that as women, the participants attested that they find it hard to disengage them from identifying with gendered norms and expectations since these actually represent their social identity.

Insight into the complex nature of well-being does not only require in-depth inner understanding. The relational dynamic between or intersection of the individual, the organization, and society (Ali et al., 2017) is important as it provides understanding of systemic conscious and unconscious behavior (Cilliers, 2017). As a result, the psycho-social dynamics uncovered in the present study, highlight how intrapersonal dynamics collude with the gendered socio-cultural and organizational system to sustain disparaging stereotypes. On the one hand, work-life balance is indeed a subjective phenomenon, which women experience in a unique way. On the other hand, it is informed collectively by socio-cultural norms, which emerge from the unconscious of the individual, the organization and society. Women's introjected beliefs and projective identification with gendered role expectations mirror and perpetuate societal and organizational dynamics in this regard.

The results show evidence of women's well-being that is impacted, however the metaphors are also images of strength and provide evidence of how women sustain their well-being. In this investigation, well-being ultimately emerged as a dynamic phenomenon that requires engagement with both positive and negative emotions and experience. According to Vleioras (2005) negative emotions trigger self-exploration leading to either more negative emotions and/or positive emotions, which further promote exploration and the development of new frames of reference. The dynamic of working with both positive and negative emotion and experience seem inevitable and important in sustaining women's well-being. This is due to a process of constant negotiation to balance the tension between the self and the idealized or social self, referred to as identity work (Adams and Crafford, 2012; Knez, 2016). Whilst the intrapersonal dynamics underlying identity work reflected women's well-being being challenged, it also reflected identity tasks applied to sustain well-being. This finding is congruent with the conclusion reached by Vleioras and Bosma (2006, p. 405) that “not dealing with identity issues is related to less psychological well-being” and “dealing with identity issues is related to more psychological well-being.” Through the SDD sessions, women were able to demonstrate how they engage with the full spectrum of inevitable positive and negative experience in identity work. In this regard, the SDD group sessions provided a space for identity tasks, in particular self-reflection and the development of self-awareness, self-authorisation, self-regard and authentic self-expression.

In this study, hope and resilience came to the fore as participants developed self-awareness of their entrenched gendered beliefs and how these influence their personal growth and confidence. Women self-authorized to be successful and thus demonstrate the potential to break free from the narrative of gendered success. By acknowledging their capacity to incorporate both male and female qualities, women also developed the potential for a self-regard that is gender neutral. They furthermore gained sight of the potential to construct a new narrative around their career identity and success. Ultimately, self-regard lead to an integrated and authentic self that “can be celebrated beyond imagination.” Seeing that authentic self-expression is crucial for optimal well-being (Waterman, 1993; Vleioras and Bosma, 2006), female employees should be provided with opportunities such as demonstrated in this study, to self-reflect on their identity work.

### Implications

Understanding well-being from a gendered perspective requires a multi-level analysis that incorporates behavioral dynamics above and below the surface of consciousness, and at the intersection of the individual, the organization, and society. Thus, an understanding of well-being is incomplete when it focuses solely on above-surface manifestations such as feeling positive, happy and satisfied. Operationalisation of SWB perspectives in research may therefore impede a fuller understanding of the phenomenon. The present study aimed to demonstrate the importance of mining unconscious experience to develop a more complex and deeper understanding of the enduring well-being dynamic. The study also demonstrated the interrelatedness of individual experience with the norms and expectations of society and the organization.

Furthermore, well-being is a complex phenomenon that cannot be conceptualized adequately as dichotomy, juxtaposing well-being with ill-being, or by negating negative experience. When working with women's well-being (in research or psychology practice) forums are necessary that will allow women to self-reflect, engaging with and integrating positive and negative experience, thoughts and feelings (*cf*. Cilliers and May, 2010). Women's well-being lies in their capability to work with both positive and negative experiences and emotions linked to their potentially conflicting work-life needs. This helps them deal with both the balance and the imbalance that result from fulfilling multiple roles, instead of focusing only on the positive (denying negative emotions) or constantly attempting to overcome negative emotions as if it is an abnormal state of ill-being (*cf*. Harris, 2009).

Well-being is related closely to identity work (*cf*. Vleioras and Bosma, 2006). This implies that doing identity work with women may facilitate their experiences of well-being during a particular career-stage. Such identity work should focus on going beyond recognizing gendered stereotypes in society and in the work context. It should create an awareness in women about their inner dynamics that collude with contextual stereotypes to sustain gendered expectations. Women can be coached to recognize how they project and introject gendered qualities and how these impact their self-worth. Ultimately, women should be guided toward self-regard. They should be helped to authorize themselves toward attaining their career needs, taking on their work-life roles and express a personal identity free from constricting gender stereotypes. The present study advances the value of action-research and demonstrates the benefits of group work (*cf*. Amon, 2017) to help women develop self-awareness, self-regard, self-authorisation, and authentic self-expression.

Employee well-being in organizations is facilitated when the work environment supports the individual's ability to think about, express and manage anxieties related to task accomplishments (Stefano et al., 2017). Therefore, implications of this study for the organization entail providing reflective space for women to do the identity work that has been described above. In providing such a space the organization will enable and facilitate female employees' self-reflection, growth, and psychological well-being. Self-reflective space could be provided explicitly through focus group forums but should also be implicit in the organizational culture.

### Limitations and directions for future research

Seeing that this study acknowledges the intersectionality of the individual, organization, and society, data was derived on an individual and group level, yet not on an organizational level. Assumptions about gendered norms and expectations on organizational and societal level, could therefore only be made to the extent that participants' gendered experiences of well-being reflect their interaction with and experience of gender dynamics within the organization and broader society. Studies providing a focus on the system psychodynamics underlying gendered work contexts as an interpretive model for studying women's well-being, continue to be needed.

To operationalise a socioanalytic inquiry is a complex task. Its methods challenged the researcher's skills for group-facilitation and required trustworthy interpretation. Being a participant-observer in SDD sessions may have limited the researcher's scope of interpretation. The researcher attempted to address this deficiency by being methodologically transparent and by clarifying the underlying meta-theoretical influences that impacted the results of this inquiry. To this end, there are boundaries to the methodology followed here. Thus, the results reflect a particular perspective to the data and the phenomenon under research; by implication, other perspectives may be possible.

Acknowledging the possibility of other perspectives highlights the need for continued similar research. Incorporating women in early and late career life-stages in similar studies may produce insightful data that can be added or compared to. For example, future researchers may explore whether gendered role expectations have shifted in the younger generations who enter the work force. The intersection of race and gender and the implications thereof have not been studied here, and will benefit from further exploration. Future research on identity work and work identity linked to well-being from a gendered perspective is also especially encouraged as a result of this study.

## Conclusion

The present study's contribution to the global body of knowledge regarding well-being from a gendered perspective, is evident in its advancing particular meta-theoretical assumptions about female employees' career needs, work-life balance and women's well-being dynamic. Meta-theoretically, the present study posits that it is inappropriate to apply conventional career theory to the study of women's work-life experiences. The more applicable alternative is to apply non-traditional, novel, and gender-specific career models, thus focusing on career needs unique to women. The study moreover refutes an over-emphasis on stereotypic work-life balance as fundamental to women's well-being.

On the one hand, the need for challenge and authentic self-expression also permeates the career journey of women and are equally important career needs in sustaining well-being. On the other hand, a gendered emphasis on work-life balance may rather have a disparaging effect on women's well-being because work-life balance is a consistent pursuit which is never fully attainable (Bloom, 2016). Over-emphasizing work-life balance in such a manner potentially creates unresolvable anxiety and pressure to be an ideally balanced individual in a social context that is fraught with work-life demands incongruent to gendered expectations. In this way, the prevailing gendered rhetoric underlying the work-life balance phenomenon is maintained. Yet, changing our deep-seated gendered perspective on work-life balance, and by implication on well-being, will prove impossible if it is not accompanied by also shifting our norms about career success. A new and different narrative of career success seems necessary to truly advance a gender neutral work-life balance phenomenon. Conceptualisation of career success should reflect a subjectivist epistemology in which the unique career needs of the individual is acknowledged and celebrated. Whilst we continue to live in a gendered society, this may however be a challenging endeavor. The value of studying well-being from a systems perspective (*cf*. Ali et al., 2017) and a psychoanalytic stance (*cf*. Stefano et al., 2017) is therefore evident. This study highlighted how women's projective identification with gendered role norms affect their well-being, yet is a result of unconscious, interrelated dynamics at the intersection of the individual, the group and the organization.

The findings contribute meta-theoretically to the discourse on women and well-being, by highlighting the value of incorporating both positive and negative experience to not only understand the challenges women face in terms of their well-being but also how they succeed in sustaining a psychological well self. In this regard, the study provides insight into a unique socioanalytic methodology in well-being research applied from a gendered perspective. Social dream drawing provides a useful mechanism to explore the deeper dynamics underpinning the difficulties that women experience in efforts to sustain their well-being. It however also provides a vehicle to facilitate important identity tasks for sustaining well-being and is proposed as one way to provide female employees with opportunities for collective self-reflection in order to develop self-regard and uncover avenues for authentic self-expression. The study demonstrates how metaphor can be applied to provide an in-depth understanding of the psycho-social dynamics that impact women's well-being as their journey continued through mid-life. The findings denoting identity work and how women sustain their well-being, may be extrapolated to a genderised work environment and applied to different similar contexts globally.

## Ethics statement

This study was carried out in accordance with the recommendations of UNISA Policy on Research Ethics and the CEMS/IOP Research Ethics Review Committee. The protocol was approved by the CEMS/IOP Research Ethics Review Committee. All subjects gave written informed consent in accordance with the Declaration of Helsinki.

## Author contributions

The author confirms being the sole contributor of this work and approved it for publication.

### Conflict of interest statement

The author declares that the research was conducted in the absence of any commercial or financial relationships that could be construed as a potential conflict of interest.

## Footnotes

^1^Long (2013), (p. xxi) prefers the term “socioanalytic,” yet recognizes that “others might use the terms system psychodynamics or psychoanalysis.” For consistency the terms socioanalytic and psychoanalytic will be used interchangeably in this study.
